# Mirroring the CANTOS revolution: is anti-inflammatory therapy for diabetes just around the corner?

**DOI:** 10.1186/s12933-017-0573-z

**Published:** 2017-07-19

**Authors:** Alexander Tenenbaum, Enrique Z. Fisman

**Affiliations:** 1 Cardiovascular Diabetology Research Foundation, 5848407 Holon, Israel; 2 Sackler Faculty of Medicine, Tel Aviv University, 6997801 Tel Aviv, Israel; 3 Cardiac Rehabilitation Institute, Sheba Medical Center, 5265601 Tel Hashomer, Israel

An awesome body of evidence supports inflammation as a pivotal player throughout all phases of diabetes and atherosclerosis development [1–3]. However, for the time being there was not any decisive substantiation that an anti-inflammatory treatment could be effective in the several clinical settings. For instance, in the SOLID-TIMI 52 trial the anti-inflammatory agent darapladib—a selective inhibitor of lipoprotein-associated phospholipase A_2_—failed to reduce the risk of recurrent coronary events [4]. On this background, the first preliminary report of phase 3 CANTOS trial results seems to be an interesting and even revolutionary game changer [5].

In this trial the monoclonal antibody canakinumab given on top of standard care significantly reduced the risk of the composite of major adverse cardiovascular events (MACE) in patients with a prior myocardial infarction and inflammatory atherosclerosis (hsCRP level ≥2 mg/L). Canakinumab works by targeting interleukin-1beta (IL-1ß), a key cytokine in the inflammatory pathway of both atherosclerosis and type 2 diabetes mellitus (T2DM) [3, 6].

In this regard, one of the first pathophysiological mechanisms involved in the initiation of low-grade systemic inflammation in T2DM, obesity and the metabolic syndrome is the anatomic and functional modification of visceral adipose tissue. Following the imbalance between energy consumption and expenditure, the gene expression profile of numerous cells is altered [7], and several dysregulated pathways [8] cause adipocytes to accumulate large amounts of fatty acids in the cytoplasm, leading to an expansive process characterized by adipocyte hyperplasia and hypertrophy.

In response to changes taking place in the structural scaffold of visceral adipose tissue, some adipocytes located in areas distant from the blood vessels undergo hypoxia and subsequent necrosis, being then surrounded by phagocytic cells. These cells re-initiate the inflammatory process via increased pro-inflammatory cytokine expression oriented to the removal of the isolated cells [9, 10]. This route is even faster when high glucose conditions are present, in which the process may be hastened by several adipocytokines inducing insulin resistance, like leptin [11] and tumor necrosis factor alfa (TNF-α) [12].

Within the above mentioned biochemical framework leading in its first stage to obesity, there is an intimate and highly coordinated association between inflammatory and metabolic pathways, highlighting so the parallel between the roles of macrophages and adipocytes [13]. Macrophages express most of the adipocyte protein products, such as fatty acid binding proteins (FABP) and peroxisome proliferator activated gamma (PPAR-γ), whereas adipocytes can express many pro-inflammatory proteins secreted mainly by macrophages, such as TNF-α and interleukin-6 (IL-6) [14]. Reduction of PPAR-γ expression and generation of a pro-inflammatory environment in white adipose tissue contributes in turn to stress-induced glucose intolerance [15]. Moreover, the functional ability of these two cell types further overlaps since macrophages can attract and store lipids to become atherosclerotic foam cells. In inflammatory conditions preadipocytes may have phagocytic and antimicrobial properties, with ability to differentiate into macrophages, suggesting a potential immunological role of preadipocytes [14, 16]. The interrelationship between adipocytes and macrophages is enhanced when there is an excess of adipose tissue, promoting thus insulin resistance [17] and eventually T2DM.

In patients with moderate or severe obesity and overt T2DM, the action of several pro-inflammatory adipocytokines like E-selectin and intercellular adhesion molecule-1 (ICAM-1) [18] closely correlates with the activation of the nuclear factor-kappa B (NFκB) [19] and IL-1β [20, 21], promoting endothelial dysfunction [22]. While adipocytes also simultaneously secrete anti-inflammatory adipocytokines like adiponectin and omentin [23], this fact is usually unable to counterbalance the detrimental inflammatory effects of the pro-inflammatory ones. Finally, the overexpression of NF-κB in adipose tissue results in a high production of damaging cytokines with concomitant aggravation of T2DM [24] and further fostering of cardiovascular complications.

Likewise the pathways described for T2DM, it has been proposed that silent subacute inflammation is also operative in type 1 diabetes mellitus (T1DM) as a promoter of cardiovascular disease development [25]. Recently, the possible role in atherosclerosis of new inflammatory biomarkers as YKL-40 has been investigated in patients with either type of diabetes [26–28]; the beneficial effects of many pharmacological and nutritional agents in diabetes, obesity and metabolic syndrome are suggestively connected to their anti-inflammatory properties in both experimental and clinical settings. Particularly, anti-inflammatory properties were demonstrated for unsaturated fatty acids [29, 30], acetylsalicylic acid [31], healthy diet [32, 33], sodium-glucose co-transporter 2 (SGLT2) inhibitors [34], glucagon-like peptide-1 (GLP-1) receptors agonists [35] and statins [36].

The CANTOS trial enrolled into the study more than 10,000 patients over the last 6 years. As previously stated, its phase 3 demonstrated that canakinumab significantly reduced the risk of MACE, a composite of cardiovascular death, and both non-fatal myocardial infarctions and strokes in patients with a prior heart attack and inflammatory atherosclerosis [5]. Thus, the anti-inflammatory agents targeting the IL-1ß pathway appear to be currently the most promising for clinicians. Specifically, dipeptidyl peptidase-4 (DPP4) inhibitors (gliptins) have been found to reduce the inflammatory state and restrain the elevation of IL-1ß in animal studies [37, 38]. The CANTOS findings reinforce the concept that atherosclerosis is a systemic disease, and in light of its inflammatory nature, it basically requires systemic therapies that can no longer be restricted to the reversal of coronary and peripheral arteries stenoses [39].

Recently, our research group has shown in a prospective randomized study that the addition of the DPP4 inhibitor vildagliptin to metformin treatment in patients with T2DM led to a significant restrain of IL-1ß levels, accompanied also with a significant reduction of hsCRP and HbA1c [40]. Therefore, seems that the entire field of inflammation and disease has reached a point where problem-oriented studies are needed to recognize specific targets for therapeutic interventions [41].

Since patients with diabetes are at a particularly high risk for cardiovascular events, the main question now is whether an appropriate anti-inflammatory strategy could be clinically effective for diabetic individuals as well. In this context, targeting inflammation to treat a given systemic disease is not nowadays “thinking outside the box”, but rather a mainstream working hypothesis. Namely, is anti-inflammatory therapy for diabetes just around the corner? Further large and well-controlled prospective clinical trials targeting inflammatory pathways for its treatment are warranted.
